# The Structure of *ampG* Gene in *Pseudomonas aeruginosa* and Its Effect on Drug Resistance

**DOI:** 10.1155/2018/7170416

**Published:** 2018-11-26

**Authors:** Qingli Chang, Chongyang Wu, Chaoqing Lin, Peizhen Li, Kaibo Zhang, Lei Xu, Yabo Liu, Junwan Lu, Cong Cheng, Qiyu Bao, Yunliang Hu, Shunfei Lu, Jinsong Li

**Affiliations:** ^1^The Second Affiliated Hospital and Yuying Children's Hospital, Wenzhou Medical University, Wenzhou 325027, China; ^2^School of Medicine and Health, Lishui University, Lishui 323000, China; ^3^School of Laboratory Medicine and Life Science, Institute of Biomedical Informatics, Wenzhou Medical University, Wenzhou 325035, China; ^4^Department of Clinical Laboratory, The First Affiliated Hospital of Xinxiang Medical University, Xinxiang 453100, China

## Abstract

In order to study the relationship between the structure and function of AmpG, structure, site-specific mutation, and gene complementary experiments have been performed against the clinical isolates of *Pseudomonas aeruginosa*. We found that there are 51 nucleotide variations at 34 loci over the *ampG* genes from 24 of 35 *P. aeruginosa* strains detected, of which 7 nucleotide variations resulted in amino acid change. The *ampG* variants with the changed nucleotides (amino acids) could complement the function of *ampG* deleted PA01 (PA01ΔG). The ampicillin minimum inhibitory concentration (MIC) of PA01ΔG complemented with 32 *ampG* variants was up to 512 *μ*g/ml, similar to the original PA01 (*P. aeruginos*a PA01). Furthermore, site-directed mutation of two conservative amino acids (I53 and W90) showed that when I53 was mutated to 53S or 53T (I53S or I53T), the ampicillin MIC level dropped drastically, and the activity of AmpC *β*-lactamase decreased as well. By contrast, the ampicillin MIC and the activity of AmpC *β*-lactamase remained unchanged for W90R and W90S mutants. Our studies demonstrated that although nucleotide variations occurred in most of the *ampG* genes, the structure of AmpG protein in clinical isolates is stable, and conservative amino acid is necessary to maintain normal function of AmpG.

## 1. Introduction

*Pseudomonas aeruginosa* is a common opportunistic pathogen and often infects people with low immunity. *P. aeruginosa* infection leads to a high fatality rate in burn patients or those needing mechanically assisted ventilation. *P. aeruginosa* also plays an important role in chronic respiratory infection, especially for those with cystic fibrosis (CF) and some other chronic respiratory system infections [1]. With the abused use of antibiotics in clinics and agriculture, some strains of *P. aeruginosa* become resistant to most if not all antibiotics, causing serious consequences especially for patients in the intensive care unit (ICU) or with chronic respiratory diseases. The main mechanism of resistance is selective mutations of the chromosome leading to a high yield of the cephalosporin lytic enzyme AmpC. *ampC* is located on the chromosome, its expression inducible by *β*-lactams, and is found in most *Enterobacteria*, *P. aeruginosa*, and other nonfermentative Gram-negative bacilli [2]. *β*-lactamases are normally expressed in low levels, but can be induced by *β*-lactam antibiotics especially cefoxitin and imipenem. The only exception is *Escherichia coli* and *Shigella* due to the lack of *ampR* [3]. Therefore, the prolonged and wide use of *β*-lactam antibiotics can result in multiple *β*-lactam-resistant bacteria that produce high levels of *β*-lactamases, causing therapeutic failures [4, 5].

The induction of AmpC production is intimately linked to peptidoglycan recycling [6]. A number of genes including *ampG*, *ampR*, *ampD*, and *ampE* are involved in the process [7]. *ampG* encodes a transmembrane protein functioning as a specific permease to transport 1,6-GldNAc-anhydro-MurNAc and 1,6-GldNAc-anhydro-MurNAc peptide, which are the signal molecules involved in *ampC* expression [8]. *ampR* encodes a DNA-binding protein belonging to the LysR superfamily [9]. There are two regulatory characteristics of *ampR*: in the absence of a *β*-lactam inducer, it binds to the UDP-MurNAc pentapeptide to promote the formation of an AmpR-DNA complex that represses *ampC* transcription, while in the presence of a *β*-lactam antibiotic, peptidoglycan fragments accumulate in the cytoplasm [10], and the 1,6-anhydro-MurNAc tripeptide (or pentapeptide) competitively displaces the UDP-MurNAc pentapeptide and converts AmpR into an activator, triggering the *ampC* expression or production of *β*-lactamase [9]. *ampD* encodes a cytosolic N-acetyl-anhydromuramyl-L-alanine amidase and specifically hydrolyzes the 1,6-anhydro-MurNAc peptide, thus inhibiting the *ampC* expression [11, 12]. *ampE* encodes a cytoplasmic membrane protein acting as a sensory transducer molecule required for *ampC* induction [13], but the exact role of AmpE is not fully understood. It has been shown that there are two *ampG* homologues in *P. aeruginosa, ampG* (PA4393) and *ampGh1* (PA4218), and only the product of *ampG* is a functional protein; the inactivation of *ampG* by mutation or deletion confers noninducible and low-level *β*-lactamase expression [14, 15]. Herein, we studied the relationship between the structure and function of *ampG* in wild type *P. aeruginosa* with different resistance levels against ampicillin. Our findings may provide more theoretical basis for identifying molecular features of AmpG and help design methods to screen candidate agents to inhibit the function of AmpG, thus prolonging the use of commonly used *β*-lactams in clinics.

## 2. Materials and Methods

### 2.1. Bacterial Strain and Plasmid

Two hundred and eleven (211) nonduplicate wild strains of *P. aeruginos*a were isolated from the clinical samples at the First Affiliated Hospital of Wenzhou Medial University, China, between 2009 and 2011. The strains were identified by the Vitek-60 microorganism autoanalysis system (BioMerieux Corporate, Lyon, France). *P. aeruginos*a PA01 and plasmids pUCP24 [14]were obtained from the Laboratory of Microbial Genetics, University of Florida, Gainesville, USA. The strains and plasmids used or constructed in this work are listed in Table 1.

### 2.2. Antibiotic Susceptibility Test

Minimal inhibitory concentrations (MICs) to the antimicrobial agents were determined by the agar dilution method for the control and recombinant strains in accordance with the guidelines of the Clinical and Laboratory Standards Institute (CLSI). Antimicrobial agents were obtained from the National Institute for the Control of Pharmaceutical and Biological Products (NICPBP) and the pharmaceutical companies in China. *E. coli* ATCC 25922 was used as a quality control for the MIC test.

### 2.3. Cloning and Comparative Analysis of the *ampG* Genes

Genomic DNA was extracted from the strains using an AxyPrep Bacterial Genomic DNA Miniprep kit (Axygen Scientific, Union City, CA, USA) and PCR- (polymerase chain reaction-) amplified to clone the *ampG* genes. The primers were designed by using Primer 5.0, and a pair of flanking restriction endonuclease adapters were added at the 5′ end of the primers (*Bam*HI for the forward primer PA_*ampG*_-F and *Hind*III for the reverse primer PA_*ampG*_-R, respectively, Table 2) and synthesized by Shanghai Sunny Biotechnology Co., Ltd (Shanghai, China). PCR amplification was carried out under the following conditions, i.e., an initial 5 min denaturation at 95°C, followed by 35 cycles of denaturation (94°C for 45 s), annealing (65°C for 45 s), and extension (72°C for 90 s), and a final extension step at 72°C for 10 min. The PCR products of *ampG*s were first cloned into a pMD18-T vector. The recombinant plasmid (pMD18-*ampG*) was identified initially by PCR and then verified by sequencing. Blast programs from NCBI (http://www.ncbi.nlm.nih.gov/BLAST) and MEGA5.05 (molecular evolutionary genetics analysis software, http://www.megasoftware.net) were used to analyze the similarities of the *ampG* nucleotide sequences and the AmpG amino acid sequences.

### 2.3. Genetic Complementation Assays to Determine the Function of the Cloned *ampG* Genes

The verified recombinant pMD18-*ampG* plasmid was digested with *Bam*HI and *Hind*III restriction enzymes. The *ampG* fragment was recovered and then ligated into a pUCP24 vector digested with the same restriction enzymes (*Bam*HI and *Hind*III). The recombinant plasmid pUCP24-*ampG* was transformed into *E. coli* JM109, and the recombinant *E. coli* JM109-pUCP24-*ampG* was further identified by PCR. pUCP24-*ampG* was extracted and introduced into PA01∆*ampG* as described previously [16]. MIC to ampicillin was performed for the recombinant PA01∆*ampG-*pUCP24-*ampG* to detect the function of the cloned *ampG*s. PA01∆*ampG* carrying the vector pUCP24 was used as a negative control.

### 2.4. Site-Directed Mutation of the Conservative Amino Acids in the *ampG* Gene

We used the *ampG* gene sequence of PA01 as the template to design primers for amplification of the 5′ and 3′ end fragments of the *ampG* gene, respectively. The forward primer for amplification of the 5′ end fragment (PA_*ampG*_-F with a *Bam*HI recognition site at the 5′ end) and the reverse primer for amplification of the 3′ end fragment (PA_*ampG*_-R with a *Hind*III recognition site at the 5′ end) were the same as described above (Table 2). The corresponding nucleotide mutations were inserted into the downstream primers of 5′ end and the upstream primer of 3′ end fragments of the *ampG* gene, and then the mixture of the PCR products of the 5′ end and the 3′ end fragments (mole ratio of 1:1) was used as the template, and the upstream primer of the 5′ end and the downstream primer of the 3′ end were used as the primers for amplification of *ampG* variants with the point mutations. Different mutations in the primers are shown in Table 2. PCRs were performed under the following conditions: an initial 5 min denaturation at 95°C followed by 35 amplification cycles, each consisting of a 40 sec denaturation step at 95°C, a 30 sec annealing step at 55°C, and a 40 sec extension step at 72°C followed by a 10 min final extension at 72°C (ExTaq from TaKaRa, Dalian, China). The PCR products (*ampG*_mut_) were purified and inserted into pMD18-T vectors. The recombinant plasmid pMD18-*ampG*_mut_ was initially identified by PCR and then verified by sequencing. The verified pMD18-*ampG*_mut_ recombinant plasmids were digested with *Bam*HI and *Hind*III. The *ampG*_mut_ fragments were recovered and ligated into the pUCP24 vectors digested with the same restriction enzymes (*Bam*HI and *Hind*III). The recombinant pUCP24-*ampG*_mut_ was then transformed into PA01∆*ampG* to detect the complementary effect of ampicillin resistance in the host.

### 2.5. Detection of *β*-Lactamase Activity

The detection procedures were performed as described in [14]. *P. aeruginosa* cells were induced for 1 h with 4 *μ*g/ml cefoxitin (Calbiochem, San Diego, USA) and for 2 h with 50 *μ*g/ml cefoxitin, respectively. Crude cell extracts were prepared by sonication, and the protein content of crude extracts was determined using BCA protein assay reagent (Pierce, USA) [17]. The *β*-lactamase activity was quantified with an UV spectrophotometer using 100 *µ*M of nitrocefin as the substrate. The activity of the *β*-lactamase was defined as nanomole of nitrocefin hydrolyzed at 30°C per min by 1 g protein. All the induction experiments were performed in triplicate, and the result represents an average of the three replicates.

## 3. Results

### 3.1. Ampicillin MIC Detection of the *P. aeruginosa* Strains

**Figure 1:**
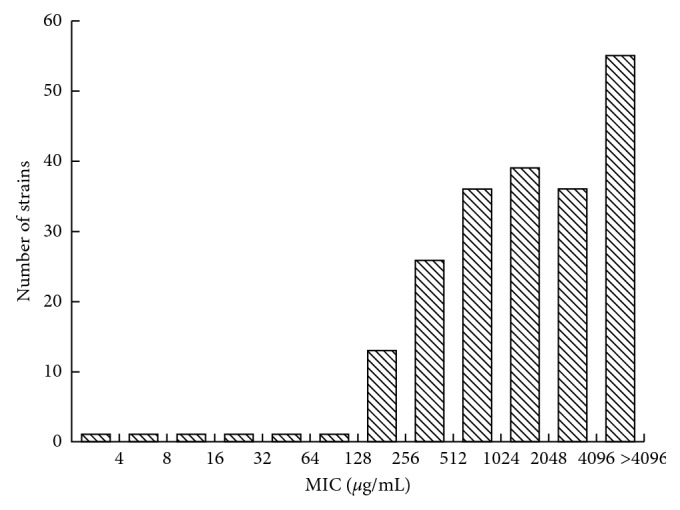
The MIC to ampicillin for 211 strains of *P. aeruginosa* had a high resistance level.

The MIC to ampicillin for 211 strains of *P. aeruginosa* showed that most of the strains had a high resistance level. Only 2.84 % (6/211) strain had a low MIC level (≤128 *μ*g/ml), and more than a half of them (61.61 %, 130/211) showed a very high MIC level of ≥2048 *μ*g/ml (Figure 1).

### 3.2. Sequence Variations of the *ampG* Genes among the Strains with Different MIC Levels

To compare the *ampG* gene structure of *P. aeruginosa* strains with different ampicillin MIC levels, we cloned and sequenced the *ampG* gene of 35 strains with high MIC levels (512 to −≥8192 *μ*g/ml). Compared with the *ampG* gene of PA01, a total of 51 nucleotide variations over 34 loci in 24 *ampG* genes were identified. Among these nucleotide variations, seven led to amino acid changes (Table 3). Three amino acids were located in the transmembrane regions, but none of them corresponded to the 51 conserved amino acids described in a recent report [18].

### 3.3. Function Analysis of *ampG* Variants from Clinically Isolated *P. aeruginosa* Strains

In order to detect the function of *ampG*s, the *ampG* genes from 24 strains with sequence variation were further cloned into pUCP24 and transformed into the PA01Δ*ampG* to perform genetic complementation analysis. All the cloned *ampG* genes complemented the function of the deleted *amp*G gene in PA01Δ*ampG*. The MIC levels for ampicillin were between 256 and 1024 *μ*g/ml, close to the level of PA01 or PA01Δ*ampG*-pUCP24-*ampG*_PA_ (512 *μ*g/ml). The AmpC *β*-lactamase activity of the genetically complemented recombinants also showed similar results (Table 3). These data indicate that the cloned *ampGs* have similar function despite their structural differences.

### 3.4. Effect of the Mutations of the Conservative Amino Acids on AmpG Function

In order to analyze the correlation of the conservative amino acids with the function of AmpG, 2 conservative amino acids I53 and W90 located in the transmembrane regions 2 and 3, respectively, were randomly chosen for site-directed mutagenesis. According to the chemical properties of the amino acids, 4 to 6 different amino acid substitutions were designed for each conserved amino acid. Finally, a total of 4 mutated genes were successfully cloned; these mutants were transformed into the recipient cell PA01Δ*ampG*; their MIC levels to ampicillin and activities of AmpC type *β*-lactamase were tested (Table 4). PA01 Δ *ampG*-pUCP24-*ampG*PA-W90R and PA01 Δ *ampG-*pUCP24-*ampG*PA-W90S had high resistance level to ampicillin, similar to that of the original *P. aeruginosa* PA01, but significantly different from that of the I53S and I53T mutants that had much lower resistance level to ampicillin and showed significantly lower AmpC type *β*-lactamase activities (Table 4).

## 4. Discussion

The clinical isolates of *P. aeruginosa* often show resistance to most antibiotics [19, 20]. The mechanism of multiple resistance is mainly related to inactivated enzyme production, overexpression of efflux systems, antibiotic target alteration, biofilm formation, and acquisition of foreign drug-resistant genes by means of horizontal gene transfer [21–23]. The inactivating enzymes to *β*-lactam antibiotics produced by *P. aeruginosa* were AmpC, the extended spectrum *β*-lactamase (ESBLs), and the metalloenzyme (MBL), of which AmpC has attracted more attention because its production can be induced [24]. Besides being encoded in *P. aeruginosa*, *ampC* extensively exists in the chromosomes or plasmids of Gram-negative bacillus such as *Enterobacter*, *Citrobacter*, and *Acinetobacter* [25, 26]. The muropeptide derivatives are essential for the induction of AmpC type *β*-lactamase expression [27]. Schmidt et al. [28] used nitrosoguanidine (NTG) to induce mutations in *E. coli* SN0301 (carrying *ampC* and *ampR* of *E. cloacae*) and obtained three mutants with mutated *ampG* genes including *ampG1* (G151D), *ampG3* (G268D), and *ampG5* (G373D), respectively. They all belong to the conserved amino acids located on the transmembrane regions 5 (158G), 10 (419G), and 13 (544G), respectively, in AmpG of *P. aeruginosa* PA01 [18]. In this work, we also performed site-directed mutation for two conserved amino acids (I53 and W59). As a result, the AmpC activity of the mutated AmpGs (I53S or I53T) located in the transmembrane regions 2 was drastically lowered. Our findings suggest that the conserved amino acids play an important role in keeping the normal function of AmpG. A single amino acid might be enough to determine the substrate specificity and the transportation activity of AmpG. The length of the AmpG proteins from different species differs from each other and certainly the transmembrane regions of the AmpGs also vary [29, 30]. A previous work from our team showed that the longest AmpG protein was from *P. aeruginosa* (CDR92618), which consists of 598 amino acids and is predicted to have 14 transmembrane regions. The shortest was from *Microcoleus* with only 401 AA and 11 transmembrane regions. Most of the AmpG proteins contain 12 or 14 transmembrane regions. Analysis of the conservative amino acid against 134 AmpGs showed that there were 51 conserved amino acids identified in 12 transmembrane regions excluding the corresponding transmembrane regions 7 and 8 of PA01. In *P. aeruginosa* PA01, transmembrane regions 1, 2, and 4 contained more conserved amino acids (with 8, 7, and 9 conserved amino acids, respectively), and transmembrane regions 6, 11, 12, and 14 contained only 1 or 2 conserved amino acids. In this work, we sequenced 35 *ampG* genes from clinically isolated *P. aeruginosa* with different MIC levels to ampicillin (512 to ≥8192 *μ*g/ml) and found 24 genes harboring 51 nucleotide variations over 34 loci. Among them, only 6 loci in 5 bacterial strains showed amino acid variations. Four strains had one amino acid difference, and one strain had 3 mutated amino acids. Amino acid change in PA01Δ*ampG*-pUCP24-*ampG28* (I439T) was located in transmembrane 11, and two of the three amino acid changes in PA01Δ*ampG*-pUCP24-*ampG*35 (T206I and G398E) were located in transmembrane regions 6 and 9, respectively. They did not belong to the 51 conserved amino acids predicted in our previous work [18]. The MIC levels for ampicillin indicated that all of these 24 genes had normal functions, suggesting that these variations both in nucleotide and amino acid sequences did not affect the function of AmpGs.

In the recent years, the clinically isolated pathogens became more and more resistant to most antibiotics. Studies on the polymorphism and relationship between the structure and function of AmpGs will help to establish a theoretical basis for the development of inhibitors against AmpG. Effective inhibitors for AmpG will shed light on prolonging the clinical use of *β*-lactam antibiotics.

## Figures and Tables

**Table 1:** Bacterial strains and plasmids.

Strains or plasmids	Relevant characteristic (s)
*Strains*	
*E. coli* JM109	endA1 hsdR17 supE44 thi-1 recA1 gyrA96 relA1 (argF-lacZYA) U169 80dlacZ
PA01	Reference strain; genome completely sequenced
PA01Δ*ampG*	*ampG* (PA4393) deleted PA01^14^

*Plasmids*	
pMD18-*ampG*_*1-35*_	pMD18 vectors carrying *ampGs* of 35 wild type *P. aeruginosa* strains, respectively (this work)
pMD18-*ampG*_*PA*_	pMD18 vector carrying *ampG* of PA01 (this work)
pUCP24	pUC18-derived broad-host-range vector; Gm^r^
pUCP24-*ampG*_*1-35*_	*ampG* genes from pMD18-*ampG1-35* cloned into pUCP24, respectively; Gmr (this work)
pUCP24-*ampG*_*PA*_	*ampG* gene of pMD18-*ampGPA* cloned into pUCP24; Gmr (this work)
pUCP24-*ampG*_*mut*_	pUCP24 vector carrying site-directedly mutated *ampG* of PA01 (this work)
pUCP24-*ampG*_PA_-I53S	
pUCP24-*ampG*_PA_-I53T	
pUCP24-*ampG*_PA_-W90R	
pUCP24-*ampG*_PA_-W90S	

**Table 2:** Primers used in this work.

Primer	Sequence^*∗*^	Purpose
PA_*ampG*_-F	5′GGGATCCCAACGCGCACGCTTGCGCGAGGA 3′(BamHI)	Cloning of *ampG* of PA01
PA_*ampG*_-R	5′GAAGCTTTCAGTGCTGCTCGGCGTTCTGGT3′(HindIII)	
PA_*ampG*_-I53S	5′CCCAGCCAACTGGCGAAACCgctGGTATCCCGCGCCACGC3′	Forward primer for I53S
PA_*ampG*_-I53T	5′CCCAGCCAACTGGCGAAACCggtGGTATCCCGCGCCACGC3′	Forward primer for I53T
PA_*ampG*_-53R	5′GGTTTCGCCAGTTGGCTGGGGCTGGTGTACGCCTTCAAGT3′	
PA_*ampG*_-W90R	5′AGCACCTGCGAGAACACCAGccgGGAACGGCGCCGGCCGA3′	Forward primer for W90R
PA_*ampG*_-W90S	5′AGCACCTGCGAGAACACCAGcgaGGAACGGCGCCGGCCGA3′	Forward primer for W90S
PA_*ampG*_-90R	5′CTGGTGTTCTCGCAGGTGCTGATCGCCCTGGGACTGCTCG3′	

^*∗*^Underlined are restriction endonuclease sites; nucleotide sequence corresponding to the mutated amino acids is shown in lowercase.

**Table 3:** Sequence variation and function of *ampGs* in clinical *P. aeruginosa* strains.

	Nucleotide		Amino acid		MIC^*∗∗*^
	Position	Variation	Position	Variation	(*μ*g/ml)
PA01Δ*ampG-*pUCP24-*ampG1*	618	C-A	206	/	512/512
PA01Δ*ampG*-pUCP24-*ampG2*	715	C-T	239	/	512/1024
	1080	G-C	360	/	
	1317	C-T	439	/	
PA01Δ*ampG*-pUCP24-*ampG3*	715	C-T	239	/	512/1024
	1080	G-C	360	/	
	1317	C-T	439	/	
PA01Δ*ampG*-pUCP24-*ampG4*	715	C-T	239	/	512/1024
	1080	G-C	360	/	
	1317	C-T	439	/	
PA01Δ*ampG*-pUCP24-*ampG7*	618	C-A	206	/	512/512
	916	G-C	306	D-H	
PA01Δ*ampG-*pUCP24-*ampG8*	27	C-A	9	/	512/1024
PA01Δ*ampG-*pUCP24-*ampG9*	27	C-A	9	/	512/512
PA01Δ*ampG-*pUCP24-*ampG10*	1329	G-A	443	/	512/1024
PA01Δ*ampG*-pUCP24-*ampG11*	666	C-T	222	/	256/512
	819	G-A	273	/	
	942	G-A	314	/	
	1062	A-G	354	/	
	1077	C-A	539	/	
	1113	C-T	371	/	
PA01Δ*ampG-*pUCP24-*ampG12*	618	C-A	206	/	512/1024
PA01Δ*ampG-*pUCP24-*ampG13*	1423	G-A	475	D-N	512/1024
PA01Δ*ampG-*pUCP24-*ampG14*	1423	G-A	475	D-N	512/1024
PA01Δ*ampG-*pUCP24-*ampG15*	336	C-T	112	/	512/4096
PA01Δ*ampG-*pUCP24-*ampG16*	1747	G-A	583	/	512/1024
PA01Δ*ampG-*pUCP24-*ampG17*	153	T-C	51	/	512/≥8192
PA01Δ*ampG-*pUCP24-*ampG20*	213	G-A	71	/	256/4096
PA01Δ*ampG-*pUCP24-*ampG24*	450	C-T	150	/	1024/4096
PA01Δ*ampG*-pUCP24-*ampG25*	336	C-T	112	/	1024/≥8192
	1027	C-T	343	/	
PA01Δ*ampG-*pUCP24-*ampG26*	1182	G-A	394	/	512/4096
PA01Δ*ampG*-pUCP24-*ampG28*	657	C-T	219	/	512/≥8192
	729	C-T	243	/	
	837	C-T	279	/	
	990	C-T	330	/	
	1062	A-G	354	/	
	1239	T-C	443	/	
	1316	T-C	439	I-T(11)^*∗*^	
PA01Δ*ampG-*pUCP24-*ampG29*	1143	C-T	381	/	512/≥8192
PA01Δ*ampG*-pUCP24-*ampG30*	27	C-A	9	/	512/≥8192
	1329	G-A	443	/	
PA01Δ*ampG*-pUCP24-*ampG31*	27	C-T	9	/	256/4096
	558	G-A	186	/	
	1002	C-T	334	/	
	1062	A-G	354	/	
	1113	C-T	371	/	
PA01Δ*ampG*-pUCP24-*ampG35*	617	C-T	206	T-I (6)^*∗*^	512/≥8192
	708	T-A	236	/	
	1193	G-A	398	G-E (9)^*∗*^	
	1364	T-C	455	L-P	
PA01Δ*ampG-*pUCP24-*ampGPA*	/	/	/	/	512
PA01Δ*ampG*	/	/	/	/	32
PA01	/	/	/	/	512
ATCC 25922	/	/	/	/	4

D: aspartic acid; H: histidine; N: asparagine; I: isoleucine; T: threonine; G: glycine; E: glutamate; L: leucine; P: proline; ^*∗*^transmembrane region; ^*∗∗*^MIC values of the cloned ORF/wild strain.

**Table 4:** MIC levels to ampicillin and AmpC *β*-lactamase activity of the site-directed mutants.

Strain	Amino acid variation	*β*-Lactamase activity	MIC (*μ*g/ml)
PA01Δ*ampG*-pUCP24-*ampG*_PA_-I53S	Ile (atc)-Ser (agc)	69.8	32
PA01Δ*ampG*-pUCP24-*ampG*_PA_-I53T	Ile (atc)-Thr (acc)	56.7	16
PA01Δ*ampG*-pUCP24-*ampG*_PA_-W90R	Try (tgg)-Arg (cgg)	25296.4	512
PA01Δ*ampG*-pUCP24-*ampG*_PA_-WA90S	Try (tgg)-Ser (tcg)	12628.9	512
PA01	/	1981.6	512
PA01Δ*ampG*	/	44.8	32
PA01Δ*ampG*-pUCP24-*ampG*_PA_	/	5019.3	1024
ATCC 25922	/	0	4

## Data Availability

The sequence variation data used to support the findings of this study are included within Table 3 in this article. The MIC levels data used to support the findings of this study are included within Table 4 in this article. Previously reported research data were used to support this study and are available at doi:10.1371/journal.pone.0168060, and these prior studies (and datasets) are cited at relevant places within the text as references [16].

## Abbreviations

ATCC: American Type Culture Collection
BLAST: Basic local alignment search tool
CF: Cystic fibrosis
CLSI: Clinical and Laboratory Standards Institute
ESBL: Extended spectrum *β*-lactamase
ICU: Intensive care unit
MIC: Minimum inhibitory concentration
NICPBP: National Institute for the Control of Pharmaceutical and Biological Products
ORF: Open reading frame
*P. aeruginosa*: *Pseudomonas aeruginosa*
PCR: Polymerase chain reaction.
